# 

**Published:** 1854-03

**Authors:** 


### NOTICES OF OUR JOURNAL.

We have received the first number of this publication, designed to aid *the people* to *prevent disease*, by proper attention to food, sleep, exercise, dress, &c. It will have a special reference to *clergymen* and *theological* Students, and as the author has for many years devoted his attention particularly to diseases of the throat and lungs, we doubt not his Journal will contain many valuable suggestions to this class. We have ourselves derived great benefit from pursuing the judicious advice given us by Dr. Hall about a year ago.—*Baltimore True Union.*

Hall's Journal of Health.—Dr. W. W. Hall, of New York, has commenced a monthly magazine with the above title. He is a physician of high standing, and has been particularly successful in treating diseases of the throat. The contents of the first number are: Editor's Address; Subjects to be Treated; Eating and Drinking; Tea and Coffee; Use of Milk; Dr. Nott's Munificence; the Food we Eat; How to attain Old Age; Too White Flour; Travelling for Health; Wearing the Beard, &c.—*Cincinnati Christ. Herald.*

"Hall's Journal of Health," is the title of a neat, well written and better edited monthly, which comes to us from New York. It is edited by Dr. W. W. Hall, a physician of varied practice, both in the South and North. This Journal will supply a want that is felt in many family circles, viz.—some plain, practical treatise which shall tell us "how to take care of health." This reminds us of what Colton says on this subject, "how happens it that all men envy us our wealth, but that no man envies us our health? The reason perhaps is that, it is very seldom that we can lose our wealth without some one being the better for it, by gaining that which we have lost; but no one is jealous of us on account of our health, because if we were to lose that, this would be a loss that betters no one." The "*Journal of Health*" before us seems to be written in a style adapted to all readers.—*West Jerseyman.*

We have received a copy of "*Dr. Hall's Journal of Health,*" and have examined the No. before us. We are favorably impressed with the lucid style in which his arguments are advanced, and think that by its general perusal a change upon the health and destinies of man might be wrought. There is great room for the improvement of the habits of the present generation, and until a change in this respect is made, disease of every type will predominate. We commend the article of "Criticus" below, who has had opportunity of discussing the merits of the work and will take pleasure in exhibiting a copy of it to all who may call at our office.

"It is the only Journal of its kind in Europe or in America. It strikes at a principle of reform not before contemplated by Medical men—'the prevention and cure of disease without medicine.' The idea at first seems to be a novel one, but we readily admit its plausibility. Dr. Hall is a regular graduate of the old school. It seems that he intends to communicate his knowledge not to the medical world, but to all mankind. The pages of the '*Health Journal*' will be devoted to this object. showing that Nature is recuperative, and capable herself of managing disease.

"We notice in the *Home Journal* several extracts from the Book lately issued by Dr. Hall, and think his extensive practice and scientific investigation commands our attention on the points above.

"A celebrated divine now living of orthodox Calvinistic faith, remarked to a class of pupils, that when the period of the Millennium came, the lives of the living would be *young* when they died at an hundred years of age.

"'How can that be?' replied the inquisitive. 'Why, sir, the reason is this, that the human constitution will be more perfectly understood, and its contingencies more naturally attended to.'"—*Chatt Jour.*

"This new Monthly is designed to instruct the community at large, but more especially Students and Professional Men in the laws of health, that by the observance of these they may forestall disease. The Editor, Dr. Hall, of this city, has had much experience in diseases of the throat and lungs, and his remarks on the causes of these will have a special value."—*New York Independent.*

"HALL'S JOURNAL OF HEALTH" assumes the principle, that "health is a duty." Dr. Hall does not give recipes, unprofessional, nor will he give a series of prosy lectures on physiology and hygiene, such as nobody will read. Dr. Hall, you are right, but the *asini multitudinum* will not profit. Teach the art of destroying life and they will build you a monument, but inculcate the art and duty of prolonging it, and they will tear down their houses to hurl brickbats at you. We are no candidate for martyrdom, yet we believe that almost all persons are suicides or are murdered.

"To those who want a good common-sense Journal of Health, without whims and technicalities, we recommend this Magazine."—*Syracuse Daily Journal.*

